# Bis[μ-1-hexyl-3-(2,3,5,6,8,9,11,12-octa­hydro-1,4,7,10,13-benzopenta­oxacyclo­penta­decin-15-yl)urea]bis­(azido­sodium) chloro­form disolvate

**DOI:** 10.1107/S1600536812015590

**Published:** 2012-04-18

**Authors:** Arnaud Gilles, Mihail Barboiu, Yves-Marie Legrand, Arie van Lee

**Affiliations:** a Institut Européen des Membranes, Université de Montpellier II, 34000 Montpellier, France

## Abstract

In the title compound, [Na_2_(N_3_)_2_(C_21_H_34_N_2_O_6_)_2_]·2CHCl_3_, the sodium cation is hepta­coordinated by five O atoms of the crown ether unit of the 1-hexyl-3-(2,3,5,6,8,9,11,12-octa­hydro-1,4,7,10,13-benzopenta­oxacyclo­penta­decin-15-yl)urea (*L*) ligand, the O atom of the urea group of the second, symmetry-related *L* ligand, and one N atom of the azide anion. The experimentally determined distance 2.472 (2) Å between the terminal azide N atom and the sodium cation is substanti­ally longer than that predicted from density functional theory (DFT) calculations (2.263 Å). The crown ethers complexing the sodium cation are related by an inversion centre and form dimers. The urea groups of the two *L* ligands are connected in a head-to-tail fashion by classical N—H⋯N hydrogen-bonding inter­actions and form a ribbon-like structure parallel to the *b* axis. Parallel ribbons are weakly linked through C—H⋯N, C—H⋯O and C—H⋯π inter­actions.

## Related literature

For the synthesis and characterization of other alkali metal azide-crown ether complexes, see: Brown *et al.* (2006 ▶, 2008 ▶). For single-crystal structure determinations of other compounds with 4-hexyl­urea-benzo-15-crown-5, see: Cazacu *et al.* (2006 ▶, 2009 ▶).
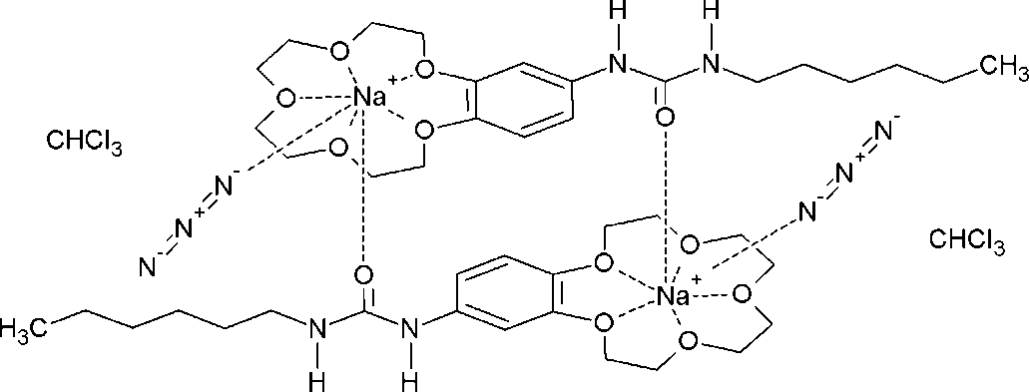


## Experimental

### 

#### Crystal data

[Na_2_(N_3_)_2_(C_21_H_34_N_2_O_6_)_2_]·2CHCl_3_
*M* *_r_* = 1189.78
Triclinic, 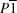

*a* = 7.8168 (3) Å
*b* = 9.9342 (3) Å
*c* = 18.5202 (7) Å
α = 82.320 (3)°
β = 83.459 (3)°
γ = 87.784 (3)°
*V* = 1415.58 (9) Å^3^
*Z* = 1
Mo *K*α radiation
μ = 0.38 mm^−1^
*T* = 175 K
0.45 × 0.20 × 0.03 mm

#### Data collection

Agilent Xcalibur Sapphire-3 CCD Gemini diffractometer
Absorption correction: multi-scan (*CrysAlis PRO*; Agilent, 2010 ▶) *T*_min_ = 0.895, *T*_max_ = 1.000
11317 measured reflections
6470 independent reflections
5176 reflections with *I* > 2σ(*I*)
*R*_int_ = 0.018

#### Refinement

*R*[*F* ^2^ > 2σ(*F* ^2^)] = 0.039
*wR*(*F* ^2^) = 0.114
*S* = 0.91
6470 reflections
340 parameters
H atoms treated by a mixture of independent and constrained refinement
Δρ_max_ = 0.58 e Å^−3^
Δρ_min_ = −0.75 e Å^−3^

### 

Data collection: *CrysAlis PRO* (Agilent, 2010 ▶); cell refinement: *CrysAlis PRO*; data reduction: *CrysAlis PRO*; program(s) used to solve structure: *SUPERFLIP* (Palatinus & Chapuis, 2007 ▶); program(s) used to refine structure: *CRYSTALS* (Betteridge *et al.*, 2003 ▶); molecular graphics: *OLEX2* (Dolomanov *et al.*, 2009 ▶); software used to prepare material for publication: *CRYSTALS*.

## Supplementary Material

Crystal structure: contains datablock(s) global, I. DOI: 10.1107/S1600536812015590/rk2333sup1.cif

Structure factors: contains datablock(s) I. DOI: 10.1107/S1600536812015590/rk2333Isup2.hkl

Additional supplementary materials:  crystallographic information; 3D view; checkCIF report

## Figures and Tables

**Table 1:** Hydrogen-bond geometry (Å, °) *Cg* is the centroid of the C16–C19/C33/C34 ring.

*D*—H⋯*A*	*D*—H	H⋯*A*	*D*⋯*A*	*D*—H⋯*A*
C2—H21⋯O23	0.96	2.46	3.244 (3)	138
C2—H21⋯N35	0.96	2.58	3.320 (3)	134
N8—H81⋯N37^i^	0.83 (2)	2.39 (3)	3.156 (3)	154 (2)
N15—H151⋯N37^i^	0.86 (2)	2.03 (2)	2.872 (3)	166 (2)
C31—H311⋯O29^ii^	0.97	2.56	3.479 (3)	159
C28—H281⋯*Cg*^ii^	0.99	2.79	3.507 (2)	131

Symmetry codes: (i) 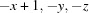; (ii) 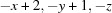.

**Fig. 1.**
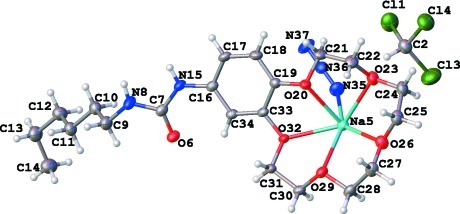
View of the title compound showing atom numbering scheme. Displacement ellipsoids are drawn at the 50% probability level. H atoms are presented as a small spheres of arbitrary radius.

**Fig. 2.**
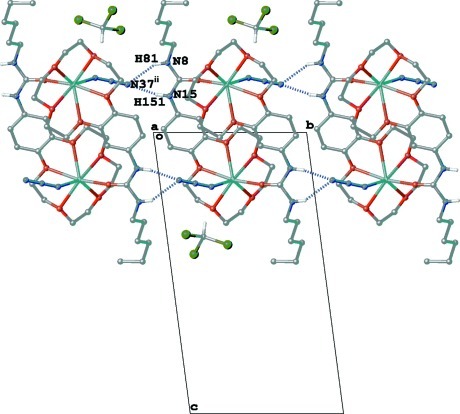
Packing of the title complex with hydrogen bonds between urea groups and azide anions indicated by dotted lines. Symmetry code: (ii) -x+1, -y, -z.

## supplementary crystallographic information

### Comment

In this contribution we present the structure of a 15 C5 crown ether (4-hexylurea-benzo-15-crown-5) co-crystallized with sodium azide and chloroform as solvate. Structures with 4-hexylurea-benzo-15-crown-5 have been recently described (Brown *et al.*, 2006; 2008). These authors focused in particular on the *M*–N_terminal_ metal-azide bond length and charge densities on the metal (*M*) and terminal nitrogen centre (N_terminal_) by X-ray crystallography and DFT calculations. They failed however to get the intended crystal structure of the coordinated sodium azide with the crown ether, obtaining only a crown ether coordination with water. In this paper, we describe the crown ether derivative, bearing a benzene ring along with a urea and hexyl alkyl chain, coordinated with sodium azide. Examples of such derivatives have been reported, but with different salts such as potassium nitrate (Cazacu *et al.*, 2009) or without any salts (Cazacu *et al.*, 2006).

In the asymmetric unit of the title compound, one 15 C5 crown ether molecules, one sodium cation, one azide molecule and one solvent chloroform molecule are present (Fig. 1). The crown ethers appear as a head-to-tail dimer, the crown ether facing the urea portion of the second molecule. Sodium is hepta-coordinated by the five oxygen atoms of the crown ether, one of the N atoms of the azide group and with the oxygen of the urea group from the second molecule. The experimental Na–N distance (2.472 (2)Å) is significantly longer than the DFT calculated Na–N distance (2.263 Å; Brown *et al.*, 2006). A possible explanation is that the azide group in the present structure is also linked through H-bonds to NH from the urea function of the second molecule, one branch being significantly stronger than the other (N15H151···N37^ii^ = 2.03 (2)Å *versus* N8H81···N37^i^ = 2.39 (3)Å). This will pull the azide group slightly outside the interaction sphere of the sodium cation. In this way coordination and H-bonds are forming a ribbon like structure along the *b*-axis (Fig. 2). The individual ribbons are weakly linked through CH···π interactions (centroid *Cg* to H281 distance 2.79Å and C28–H281···*Cg* angle 131°, where *Cg* is the centroid formed by the aromatic ring C16-C19/C33/C34). A weak CH···O non-classical interaction is present between C31 and O29 - C31H311···O29^iii^ = 2.56Å. Symmetry codes: (ii) -*x*+1, -*y*, -*z*;(iii) -*x*+2, -*y*+1, -*z*.

### Experimental

To a solution of 4'-aminobenzo-15-crown-5 (300 mg, 1.06 mmol, 1eq.) in acetonitrile was added hexyl isocyanate (175 mg, 1.38 mmol, 1.3 eq.). After 12 h under reflux, the solution was evaporated. The residue was dissolved in chloroform and precipitated by addition of hexane. The resulting precipitate was isolated by filtration and washed with hexane to give the crude title compound (302 mg, 70% yield). The compound was dissolved in chloroform, a small amount of NaN_3_ was added and the mixture was sonicated for 30 minutes. The compound crystallized after a few hours.

### Refinement

The H atoms were all located in a difference map, but those attached to carbon atoms were repositioned geometrically. The H atoms attached to carbon atoms were initially refined with soft restraints on the bond lengths and angles to regularize their geometry - C–H in the range 0.93Å-0.98Å) and *U*_iso_(H) = 1.2(1.5)*U*_eq_(C), after which the positions were refined with riding constraints. The positions of the H atoms attached to nitrogen atoms were freely refined, but their isotropic atomic displacement parameter were constrained as for the other H atoms.

### Crystal data

**Table:**

[Na_2_(N_3_)_2_(C_21_H_34_N_2_O_6_)_2_]·2CHCl_3_	*Z* = 1
*M**_r_* = 1189.78	*F*(000) = 624
Triclinic, *P*1	*D*_x_ = 1.396 Mg m^−^^3^
Hall symbol: -P 1	Mo *K*α radiation, λ = 0.71073 Å
*a* = 7.8168 (3) Å	Cell parameters from 3569 reflections
*b* = 9.9342 (3) Å	θ = 2.1–29.0°
*c* = 18.5202 (7) Å	µ = 0.38 mm^−^^1^
α = 82.320 (3)°	*T* = 175 K
β = 83.459 (3)°	Needle, colourless
γ = 87.784 (3)°	0.45 × 0.20 × 0.03 mm
*V* = 1415.58 (9) Å^3^	

### Data collection

**Table:**

Agilent Xcalibur Sapphire-3 CCD Gemini diffractometer	6470 independent reflections
Radiation source: Enhance (Mo) X-ray Source	5176 reflections with *I* > 2σ(*I*)
Graphite monochromator	*R*_int_ = 0.018
Detector resolution: 16.0143 pixels mm^-1^	θ_max_ = 29.1°, θ_min_ = 2.1°
ω scans	*h* = −10→6
Absorption correction: multi-scan (*CrysAlis PRO*; Agilent, 2010)	*k* = −12→13
*T*_min_ = 0.895, *T*_max_ = 1.000	*l* = −21→24
11317 measured reflections	

### Refinement

**Table:**

Refinement on *F*^2^	Primary atom site location: structure-invariant direct methods
Least-squares matrix: full	Secondary atom site location: difference Fourier map
*R*[*F*^2^ > 2σ(*F*^2^)] = 0.039	Hydrogen site location: inferred from neighbouring sites
*wR*(*F*^2^) = 0.114	H atoms treated by a mixture of independent and constrained refinement
*S* = 0.91	Modified Sheldrick method *w* = 1/[σ^2^(*F*^2^) + (0.07*P*)^2^ + 0.69*P*], where *P* = (max(*F*_o_^2^,0) + 2*F*_c_^2^)/3
6470 reflections	(Δ/σ)_max_ < 0.001
340 parameters	Δρ_max_ = 0.58 e Å^−^^3^
0 restraints	Δρ_min_ = −0.75 e Å^−^^3^

### Fractional atomic coordinates and isotropic or equivalent isotropic displacement parameters (Å^2^)

**Table:**

	*x*	*y*	*z*	*U*_iso_*/*U*_eq_	
Cl1	0.29314 (7)	0.12610 (6)	0.36316 (4)	0.0548	
C2	0.4561 (2)	0.23861 (19)	0.37104 (11)	0.0356	
Cl3	0.36653 (10)	0.38944 (6)	0.39926 (4)	0.0680	
Cl4	0.59472 (7)	0.16173 (6)	0.43316 (3)	0.0512	
H21	0.5200	0.2581	0.3235	0.0434*	
Na5	0.68726 (8)	0.47918 (6)	0.16960 (4)	0.0242	
O6	0.48814 (15)	0.33622 (11)	−0.18581 (7)	0.0285	
C7	0.5384 (2)	0.21761 (16)	−0.18696 (10)	0.0242	
N8	0.5406 (2)	0.15234 (17)	−0.24686 (9)	0.0374	
C9	0.4602 (3)	0.20770 (19)	−0.31162 (11)	0.0355	
C10	0.2949 (2)	0.13728 (18)	−0.31851 (10)	0.0313	
C11	0.2103 (2)	0.19461 (19)	−0.38655 (10)	0.0333	
C12	0.0434 (3)	0.1250 (2)	−0.39224 (12)	0.0389	
C13	−0.0472 (3)	0.1801 (2)	−0.45926 (12)	0.0418	
C14	−0.1142 (3)	0.3249 (2)	−0.45882 (13)	0.0478	
H143	−0.1799	0.3539	−0.4990	0.0713*	
H142	−0.0221	0.3863	−0.4614	0.0704*	
H141	−0.1882	0.3325	−0.4137	0.0719*	
H132	0.0331	0.1764	−0.5028	0.0512*	
H131	−0.1463	0.1226	−0.4624	0.0506*	
H122	−0.0352	0.1330	−0.3482	0.0473*	
H121	0.0706	0.0298	−0.3933	0.0470*	
H112	0.2902	0.1861	−0.4306	0.0398*	
H111	0.1888	0.2919	−0.3852	0.0400*	
H102	0.2142	0.1441	−0.2755	0.0370*	
H101	0.3182	0.0418	−0.3200	0.0371*	
H92	0.5433	0.1998	−0.3547	0.0427*	
H91	0.4372	0.3008	−0.3078	0.0431*	
N15	0.59760 (19)	0.13769 (14)	−0.12871 (8)	0.0269	
C16	0.64335 (19)	0.17637 (15)	−0.06340 (9)	0.0214	
C17	0.6957 (2)	0.07199 (15)	−0.01212 (10)	0.0249	
C18	0.7524 (2)	0.10018 (15)	0.05227 (9)	0.0258	
C19	0.7575 (2)	0.23293 (15)	0.06711 (9)	0.0218	
O20	0.82203 (15)	0.27206 (11)	0.12686 (6)	0.0260	
C21	0.8591 (3)	0.16670 (17)	0.18375 (10)	0.0334	
C22	0.9369 (3)	0.23127 (19)	0.24011 (11)	0.0349	
O23	0.81354 (16)	0.32292 (13)	0.27110 (7)	0.0309	
C24	0.8829 (3)	0.3975 (2)	0.32158 (10)	0.0346	
C25	1.0045 (2)	0.50500 (19)	0.28352 (10)	0.0335	
O26	0.91448 (15)	0.58835 (12)	0.23138 (7)	0.0299	
C27	1.0246 (2)	0.68301 (18)	0.18535 (10)	0.0306	
C28	0.9298 (2)	0.74580 (16)	0.12253 (10)	0.0268	
O29	0.89393 (14)	0.64030 (11)	0.08208 (6)	0.0247	
C30	0.8041 (2)	0.68851 (15)	0.02080 (9)	0.0224	
C31	0.7725 (2)	0.57005 (15)	−0.01825 (9)	0.0220	
O32	0.69444 (14)	0.46654 (10)	0.03651 (6)	0.0217	
C33	0.69806 (19)	0.33699 (14)	0.01680 (9)	0.0193	
C34	0.64267 (19)	0.31041 (15)	−0.04782 (9)	0.0209	
H341	0.6064	0.3812	−0.0801	0.0243*	
H311	0.8789	0.5349	−0.0418	0.0259*	
H312	0.6931	0.5978	−0.0562	0.0255*	
H302	0.6927	0.7308	0.0380	0.0260*	
H301	0.8754	0.7532	−0.0129	0.0261*	
H282	0.9976	0.8153	0.0910	0.0316*	
H281	0.8213	0.7896	0.1414	0.0311*	
H272	1.1304	0.6344	0.1671	0.0363*	
H271	1.0565	0.7540	0.2137	0.0370*	
H252	1.1098	0.4676	0.2585	0.0386*	
H251	1.0413	0.5567	0.3199	0.0395*	
H242	0.9400	0.3365	0.3574	0.0420*	
H241	0.7839	0.4424	0.3465	0.0405*	
H222	0.9731	0.1600	0.2775	0.0429*	
H221	1.0366	0.2786	0.2178	0.0406*	
H212	0.7521	0.1202	0.2038	0.0389*	
H211	0.9414	0.1031	0.1638	0.0403*	
H181	0.7864	0.0271	0.0860	0.0310*	
H171	0.6928	−0.0178	−0.0217	0.0294*	
N35	0.4334 (2)	0.33243 (16)	0.19341 (10)	0.0391	
N36	0.38153 (18)	0.22991 (14)	0.18164 (8)	0.0282	
N37	0.3246 (3)	0.12827 (17)	0.17006 (11)	0.0481	
H81	0.575 (3)	0.072 (3)	−0.2411 (14)	0.0500*	
H151	0.620 (3)	0.054 (3)	−0.1333 (14)	0.0500*	

### Atomic displacement parameters (Å^2^)

**Table:**

	*U*^11^	*U*^22^	*U*^33^	*U*^12^	*U*^13^	*U*^23^
Cl1	0.0435 (3)	0.0650 (4)	0.0582 (4)	−0.0145 (3)	−0.0086 (3)	−0.0103 (3)
C2	0.0341 (10)	0.0398 (10)	0.0324 (10)	0.0002 (7)	−0.0050 (8)	−0.0023 (8)
Cl3	0.0774 (5)	0.0451 (3)	0.0827 (5)	0.0180 (3)	−0.0132 (4)	−0.0146 (3)
Cl4	0.0505 (3)	0.0560 (3)	0.0489 (3)	0.0066 (2)	−0.0208 (2)	−0.0026 (2)
Na5	0.0251 (3)	0.0210 (3)	0.0265 (3)	0.0023 (2)	−0.0035 (3)	−0.0037 (2)
O6	0.0289 (6)	0.0231 (6)	0.0352 (7)	0.0056 (4)	−0.0089 (5)	−0.0076 (5)
C7	0.0184 (7)	0.0236 (8)	0.0321 (9)	−0.0002 (6)	−0.0042 (6)	−0.0074 (6)
N8	0.0505 (10)	0.0286 (8)	0.0380 (9)	0.0108 (7)	−0.0195 (8)	−0.0134 (7)
C9	0.0438 (11)	0.0340 (9)	0.0313 (10)	−0.0015 (8)	−0.0114 (8)	−0.0070 (7)
C10	0.0341 (10)	0.0335 (9)	0.0264 (9)	0.0031 (7)	−0.0035 (7)	−0.0054 (7)
C11	0.0371 (10)	0.0356 (10)	0.0279 (9)	0.0000 (7)	−0.0082 (8)	−0.0036 (7)
C12	0.0429 (11)	0.0351 (10)	0.0400 (11)	−0.0029 (8)	−0.0135 (9)	−0.0016 (8)
C13	0.0465 (12)	0.0432 (11)	0.0391 (11)	−0.0019 (9)	−0.0163 (9)	−0.0080 (8)
C14	0.0553 (14)	0.0410 (11)	0.0503 (13)	0.0002 (9)	−0.0238 (11)	−0.0032 (9)
N15	0.0322 (8)	0.0185 (6)	0.0325 (8)	0.0029 (5)	−0.0095 (6)	−0.0083 (5)
C16	0.0166 (7)	0.0198 (7)	0.0281 (9)	0.0000 (5)	−0.0013 (6)	−0.0052 (6)
C17	0.0255 (8)	0.0154 (7)	0.0342 (9)	−0.0003 (6)	−0.0029 (7)	−0.0045 (6)
C18	0.0290 (9)	0.0175 (7)	0.0297 (9)	0.0023 (6)	−0.0040 (7)	0.0015 (6)
C19	0.0211 (7)	0.0207 (7)	0.0230 (8)	0.0011 (5)	−0.0013 (6)	−0.0018 (6)
O20	0.0355 (6)	0.0193 (5)	0.0235 (6)	0.0033 (4)	−0.0085 (5)	−0.0009 (4)
C21	0.0457 (11)	0.0228 (8)	0.0319 (10)	0.0072 (7)	−0.0139 (8)	0.0017 (7)
C22	0.0406 (10)	0.0310 (9)	0.0346 (10)	0.0115 (7)	−0.0151 (8)	−0.0040 (7)
O23	0.0302 (6)	0.0373 (7)	0.0258 (6)	0.0011 (5)	−0.0063 (5)	−0.0045 (5)
C24	0.0383 (10)	0.0424 (10)	0.0245 (9)	0.0026 (8)	−0.0078 (7)	−0.0063 (7)
C25	0.0298 (9)	0.0417 (10)	0.0319 (10)	0.0023 (7)	−0.0114 (7)	−0.0091 (8)
O26	0.0224 (6)	0.0370 (7)	0.0298 (7)	−0.0016 (5)	−0.0035 (5)	−0.0024 (5)
C27	0.0218 (8)	0.0374 (9)	0.0337 (10)	−0.0064 (7)	−0.0013 (7)	−0.0087 (7)
C28	0.0229 (8)	0.0242 (8)	0.0346 (10)	−0.0043 (6)	−0.0005 (7)	−0.0092 (7)
O29	0.0251 (6)	0.0207 (5)	0.0300 (6)	0.0021 (4)	−0.0068 (5)	−0.0069 (4)
C30	0.0213 (8)	0.0167 (7)	0.0287 (9)	0.0009 (5)	−0.0020 (6)	−0.0017 (6)
C31	0.0258 (8)	0.0173 (7)	0.0218 (8)	0.0005 (5)	−0.0017 (6)	0.0003 (6)
O32	0.0287 (6)	0.0140 (5)	0.0221 (6)	0.0003 (4)	−0.0011 (4)	−0.0023 (4)
C33	0.0178 (7)	0.0154 (7)	0.0240 (8)	0.0013 (5)	0.0005 (6)	−0.0030 (5)
C34	0.0174 (7)	0.0179 (7)	0.0270 (8)	0.0022 (5)	−0.0023 (6)	−0.0025 (6)
N35	0.0396 (9)	0.0360 (9)	0.0428 (10)	−0.0108 (7)	−0.0045 (7)	−0.0059 (7)
N36	0.0255 (7)	0.0263 (7)	0.0318 (8)	0.0053 (5)	−0.0007 (6)	−0.0041 (6)
N37	0.0555 (11)	0.0273 (8)	0.0625 (13)	−0.0011 (7)	0.0042 (9)	−0.0189 (8)

### Geometric parameters (Å, º)

**Table:**

Cl1—C2	1.758 (2)	C17—H171	0.934
C2—Cl3	1.747 (2)	C18—C19	1.385 (2)
C2—Cl4	1.751 (2)	C18—H181	0.943
C2—H21	0.962	C19—O20	1.374 (2)
Na5—O6^i^	2.2786 (12)	C19—C33	1.399 (2)
Na5—O20	2.4592 (12)	O20—C21	1.4284 (19)
Na5—O23	2.5380 (14)	C21—C22	1.492 (3)
Na5—O26	2.5658 (13)	C21—H212	0.981
Na5—O29	2.5902 (13)	C21—H211	0.963
Na5—O32	2.4790 (13)	C22—O23	1.426 (2)
Na5—N35	2.4718 (17)	C22—H222	0.979
O6—C7	1.2298 (19)	C22—H221	0.950
C7—N8	1.356 (2)	O23—C24	1.432 (2)
C7—N15	1.365 (2)	C24—C25	1.505 (3)
N8—C9	1.450 (2)	C24—H242	0.973
N8—H81	0.83 (2)	C24—H241	0.979
C9—C10	1.519 (3)	C25—O26	1.417 (2)
C9—H92	0.979	C25—H252	0.983
C9—H91	0.946	C25—H251	0.973
C10—C11	1.522 (2)	O26—C27	1.426 (2)
C10—H102	0.966	C27—C28	1.503 (3)
C10—H101	0.963	C27—H272	0.990
C11—C12	1.521 (3)	C27—H271	0.989
C11—H112	0.980	C28—O29	1.4209 (19)
C11—H111	0.978	C28—H282	0.974
C12—C13	1.526 (3)	C28—H281	0.988
C12—H122	0.975	O29—C30	1.4216 (19)
C12—H121	0.964	C30—C31	1.503 (2)
C13—C14	1.513 (3)	C30—H302	0.990
C13—H132	0.967	C30—H301	0.977
C13—H131	0.992	C31—O32	1.4446 (18)
C14—H143	0.959	C31—H311	0.969
C14—H142	0.954	C31—H312	0.995
C14—H141	0.970	O32—C33	1.3823 (17)
N15—C16	1.404 (2)	C33—C34	1.379 (2)
N15—H151	0.86 (2)	C34—H341	0.918
C16—C17	1.392 (2)	N35—N36	1.166 (2)
C16—C34	1.399 (2)	N36—N37	1.174 (2)
C17—C18	1.384 (2)		
			
Cl1—C2—Cl3	110.46 (11)	C18—C17—H171	120.1
Cl1—C2—Cl4	109.86 (11)	C17—C18—C19	120.63 (15)
Cl3—C2—Cl4	110.45 (11)	C17—C18—H181	118.6
Cl1—C2—H21	107.5	C19—C18—H181	120.7
Cl3—C2—H21	109.4	C18—C19—O20	125.05 (14)
Cl4—C2—H21	109.1	C18—C19—C33	118.40 (15)
O6^i^—Na5—O20	164.07 (5)	O20—C19—C33	116.52 (13)
O6^i^—Na5—O23	125.34 (5)	Na5—O20—C19	114.99 (9)
O20—Na5—O23	67.50 (4)	Na5—O20—C21	114.92 (10)
O6^i^—Na5—O26	88.32 (4)	C19—O20—C21	116.83 (12)
O20—Na5—O26	106.72 (4)	O20—C21—C22	107.44 (14)
O23—Na5—O26	65.51 (4)	O20—C21—H212	109.0
O6^i^—Na5—O29	87.68 (4)	C22—C21—H212	112.3
O20—Na5—O29	93.92 (4)	O20—C21—H211	109.2
O23—Na5—O29	117.85 (4)	C22—C21—H211	109.0
O26—Na5—O29	64.89 (4)	H212—C21—H211	109.9
O6^i^—Na5—O32	102.87 (5)	C21—C22—O23	109.09 (15)
O20—Na5—O32	64.24 (4)	C21—C22—H222	108.9
O23—Na5—O32	131.59 (4)	O23—C22—H222	111.5
O26—Na5—O32	125.91 (4)	C21—C22—H221	109.3
O29—Na5—O32	62.98 (4)	O23—C22—H221	110.2
O6^i^—Na5—N35	89.71 (5)	H222—C22—H221	107.7
O20—Na5—N35	81.26 (5)	C22—O23—Na5	109.80 (10)
O23—Na5—N35	86.68 (5)	C22—O23—C24	112.56 (14)
O26—Na5—N35	143.72 (6)	Na5—O23—C24	111.84 (10)
O29—Na5—N35	151.19 (6)	O23—C24—C25	112.33 (15)
O32—Na5—N35	89.77 (5)	O23—C24—H242	110.7
Na5^i^—O6—C7	161.14 (11)	C25—C24—H242	110.3
O6—C7—N8	123.10 (16)	O23—C24—H241	105.6
O6—C7—N15	124.07 (15)	C25—C24—H241	108.4
N8—C7—N15	112.83 (14)	H242—C24—H241	109.4
C7—N8—C9	123.28 (16)	C24—C25—O26	107.30 (14)
C7—N8—H81	114.7 (18)	C24—C25—H252	113.3
C9—N8—H81	121.2 (18)	O26—C25—H252	109.3
N8—C9—C10	112.91 (16)	C24—C25—H251	108.9
N8—C9—H92	108.2	O26—C25—H251	111.7
C10—C9—H92	110.2	H252—C25—H251	106.4
N8—C9—H91	106.4	C25—O26—Na5	118.39 (10)
C10—C9—H91	110.2	C25—O26—C27	111.97 (13)
H92—C9—H91	108.9	Na5—O26—C27	116.52 (10)
C9—C10—C11	113.14 (16)	O26—C27—C28	108.37 (13)
C9—C10—H102	109.7	O26—C27—H272	109.0
C11—C10—H102	109.3	C28—C27—H272	110.5
C9—C10—H101	109.7	O26—C27—H271	109.7
C11—C10—H101	108.4	C28—C27—H271	110.2
H102—C10—H101	106.3	H272—C27—H271	109.0
C10—C11—C12	112.71 (16)	C27—C28—O29	107.72 (13)
C10—C11—H112	109.9	C27—C28—H282	111.4
C12—C11—H112	109.3	O29—C28—H282	110.2
C10—C11—H111	108.1	C27—C28—H281	109.9
C12—C11—H111	110.2	O29—C28—H281	110.0
H112—C11—H111	106.5	H282—C28—H281	107.6
C11—C12—C13	114.65 (17)	C28—O29—Na5	106.07 (9)
C11—C12—H122	109.0	C28—O29—C30	112.59 (12)
C13—C12—H122	108.9	Na5—O29—C30	106.28 (9)
C11—C12—H121	107.3	O29—C30—C31	108.55 (12)
C13—C12—H121	109.3	O29—C30—H302	109.4
H122—C12—H121	107.4	C31—C30—H302	109.7
C12—C13—C14	113.81 (17)	O29—C30—H301	109.2
C12—C13—H132	108.7	C31—C30—H301	108.5
C14—C13—H132	108.2	H302—C30—H301	111.3
C12—C13—H131	109.8	C30—C31—O32	106.77 (12)
C14—C13—H131	107.7	C30—C31—H311	111.4
H132—C13—H131	108.4	O32—C31—H311	109.9
C13—C14—H143	112.0	C30—C31—H312	110.3
C13—C14—H142	111.2	O32—C31—H312	109.9
H143—C14—H142	108.0	H311—C31—H312	108.6
C13—C14—H141	109.7	C31—O32—Na5	122.13 (9)
H143—C14—H141	108.0	C31—O32—C33	115.81 (11)
H142—C14—H141	107.8	Na5—O32—C33	115.59 (9)
C7—N15—C16	128.58 (14)	C19—C33—O32	116.18 (14)
C7—N15—H151	117.3 (17)	C19—C33—C34	121.49 (13)
C16—N15—H151	113.8 (17)	O32—C33—C34	122.31 (13)
N15—C16—C17	116.26 (14)	C16—C34—C33	119.62 (14)
N15—C16—C34	124.69 (15)	C16—C34—H341	121.0
C17—C16—C34	119.04 (15)	C33—C34—H341	119.3
C16—C17—C18	120.72 (14)	Na5—N35—N36	143.11 (14)
C16—C17—H171	119.2	N35—N36—N37	178.08 (19)

Symmetry code: (i) −*x*+1, −*y*+1, −*z*.

### Hydrogen-bond geometry (Å, º)

Cg is the centroid of the C16–C19/C33/C34 ring.

**Table:**

*D*—H···*A*	*D*—H	H···*A*	*D*···*A*	*D*—H···*A*
C2—H21···O23	0.96	2.46	3.244 (3)	138
C2—H21···N35	0.96	2.58	3.320 (3)	134
N8—H81···N37^ii^	0.83 (2)	2.39 (3)	3.156 (3)	154 (2)
N15—H151···N37^ii^	0.86 (2)	2.03 (2)	2.872 (3)	166 (2)
C31—H311···O29^iii^	0.97	2.56	3.479 (3)	159
C28—H281···*Cg*^iii^	0.99	2.79	3.507 (2)	131

Symmetry codes: (ii) −*x*+1, −*y*, −*z*; (iii) −*x*+2, −*y*+1, −*z*.
